# Interaponeurosis shear strain modulates behavior of myotendinous junction of the human triceps surae

**DOI:** 10.1002/phy2.147

**Published:** 2013-11-07

**Authors:** Ryuta Kinugasa, Toshiaki Oda, Toshihiko Komatsu, V Reggie Edgerton, Shantanu Sinha

**Affiliations:** 1 Department of Human Sciences, Kanagawa UniversityKanagawa, Japan; 2 Imaging Processing Research Team, Center for Advanced Photonics, RIKENSaitama, Japan; 3 Robot Sensor Systems Research Team, RIKEN-TRI Collaboration Center for Human-Interactive Robot ResearchAichi, Japan; 4 Human Life and Health Sciences, Hyogo University of Teacher EducationHyogo, Japan; 5 Center for Education in Liberal Arts and Sciences, Osaka UniversityOsaka, Japan; 6 Department of Integrative Biology and Physiology, University of CaliforniaLos Angeles, California; 7 Department of Neurobiology, University of CaliforniaLos Angeles, California; 8 Brain Research Institute, University of CaliforniaLos Angeles, California; 9 Department of Radiology, University of CaliforniaSan Diego, California

**Keywords:** Human, interaponeurosis shear strain, myotendinous junction, skeletal muscle

## Abstract

Muscle fascicles insert into a sheet-like aponeurosis. Adjacent aponeuroses are structurally in contact with each other, and ultimately merge into a common tendon. Consequently, fascicle shortening in planes of tissue layers in adjacent compartments must cause sliding between aponeuroses parallel to the acting forces. In this study, we used velocity-encoded, phase-contrast, and water-saturated spin-lattice relaxation time-weighted imaging to identify and track fascicle and aponeurosis behaviors of human medial gastrocnemius (MG) and soleus (Sol) during 15° dorsiflexion to 30° plantarflexion contractions of the ankle. Interaponeurosis shear strain, which was defined as the relative displacement of the aponeurosis at the fascicle end points (insertion) of the MG and Sol, was an average of 1.35 ± 0.27% (range 1.12 ∼ 1.87%), indicating that the strain is greater in the aponeurosis of MG fascicle insertion than the Sol. The myotendinous junction (MTJ) displacement increased significantly with decreasing interaponeurosis shear strain (*P* < 0.05). The magnitude of interaponeurosis shear strain had significant correlation with the temporal difference between the time at which the peak aponeurosis displacement of the MG and Sol occurred (*P* < 0.05). Our model also indicated that theoretical MTJ displacement varies in relation to temporal difference: no temporal difference caused the largest MTJ displacement and presence of temporal differences indicated a reduction in MTJ displacement. Therefore, we concluded that interaponeurosis shear strain is a mechanism enabling individual muscle contraction and thus specific loading of the tendon and joint.

## Introduction

In several skeletal muscles, the force-generating fibers are oriented at an angle relative to the muscle's line of action (Gans 1982). When fibers in a pinnate muscle shorten, they rotate to greater angles of pennation (Fukunaga et al. 1997). Consequently, fiber length changes translate into the movement of the aponeurosis along its line of action (Hodgson et al. 2006; Azizi and Brainerd 2008). Aponeuroses serve as the origin and insertion surfaces of muscle fascicles and therefore cover a substantial portion of the muscle belly. An important feature of this musculoskeletal structure is that some aponeuroses merge into a common tendon and which ultimately inserts on to the bone. Therefore, the cumulative result of the displacement of multiple aponeuroses along the muscles’ line of action may be to dynamically increase tendon strain and thus have important implications for the capacity of total force output. From another perspective, one can also regard such intricate interplay between the anatomical design and contractile properties of the muscle, aponeurosis, and tendons as mechanisms for allowing requisite adjustments of these parameters in order to produce at the end optimal function in terms of force or movement, either in normal conditions or diseased.

In contrast, it is generally recognized that an aponeurosis may connect structurally over a common surface with another adjacent aponeurosis (Hodgson et al. 2006; Kinugasa et al. 2008). Our group has reported that the aponeurosis of the soleus (Sol) fascicle insertion appeared to be separated from the aponeurosis of gastrocnemius insertion over most of their lengths in the visible human data (Hodgson et al. 2006). Additionally, dissection of the aponeuroses from the Sol of two cadavers revealed that the Sol and gastrocnemius aponeuroses were for the most part separate, but fused over the most distal 5 cm of the Sol muscle (Hodgson et al. 2006). The coupling of adjacent aponeuroses has the potential to generate a loading regime that is more complex than that generated in the common tendon. A previous study has in fact observed differential strain occurring between adjacent aponeuroses (Bojsen-Møller et al. 2004). Because the actions of multiple aponeuroses finally determine the strain of the myotendinous junction (MTJ) and ultimately the tendon strain, the nature of the coupling between these two adjacent aponeuroses, belonging to two separate muscles but in contact with each other, is undoubtedly important. However, the relationship between interaponeurosis shear strain and MTJ behavior has been largely unexplored.

Because of the differences in contraction time (Harridge et al. 1996) and fiber shortening velocities (Chino et al. 2006) between the predominantly slow Sol and mixed fiber-type content of the medial gastrocnemius (MG) (Edgerton et al. 1975), different levels of interaponeurosis shear strain can be expected, resulting in a temporal difference in the contractile events, that is, timing of the greatest displacement mismatch between the MG and Sol muscles. We therefore hypothesized that the geometric relationship between the aponeurosis of the MG and Sol fascicles insertion and differences in the timing of force development between two muscles can result in shear strain in adjacent aponeuroses which may in turn impact the displacement of the MTJ. The MTJ in this study was defined as the distal portion of the MG and the point at which the aponeurosis of the Sol fascicle insertion connects and finally merges with the Achilles tendon. Toward this end, our objective in this investigation was to determine empirically the effect of interaponeurosis shear strain between the aponeurosis of the MG and Sol fascicles insertion on the MTJ displacement in a contracting muscle using phase-contrast, velocity-encoded (VE-PC) magnetic resonance imaging (MRI) and compare the results with predictions from theoretical modeling. Furthermore, an in vitro pilot study was performed with cadaveric dissection to investigate the intrinsic connective tissue structures between the aponeurosis of the MG and Sol fascicles insertion. In an attempt to better understand the principles determining the effect of a wide variety of temporal differences on the behavior of the MTJ, we supplemented our experimental investigative efforts with theoretical modeling.

## Methods

### In vitro study

The in vitro cadaveric pilot study was approved by the ethical review board of Osaka University. A calf muscle from a full-body cadaver was obtained. Specimens chosen for the study showed no evidence of having had previous surgical intervention in the posterior compartment of the leg. The skin, subcutaneous tissue, and most superficial fascia were removed from the specimens to expose the entire triceps surae. The most proximal muscular attachments of both gastrocnemius heads were transected at the level of the knee. The connectivity of distal adjacent aponeuroses between MG and Sol was identified.

**Figure 1:**
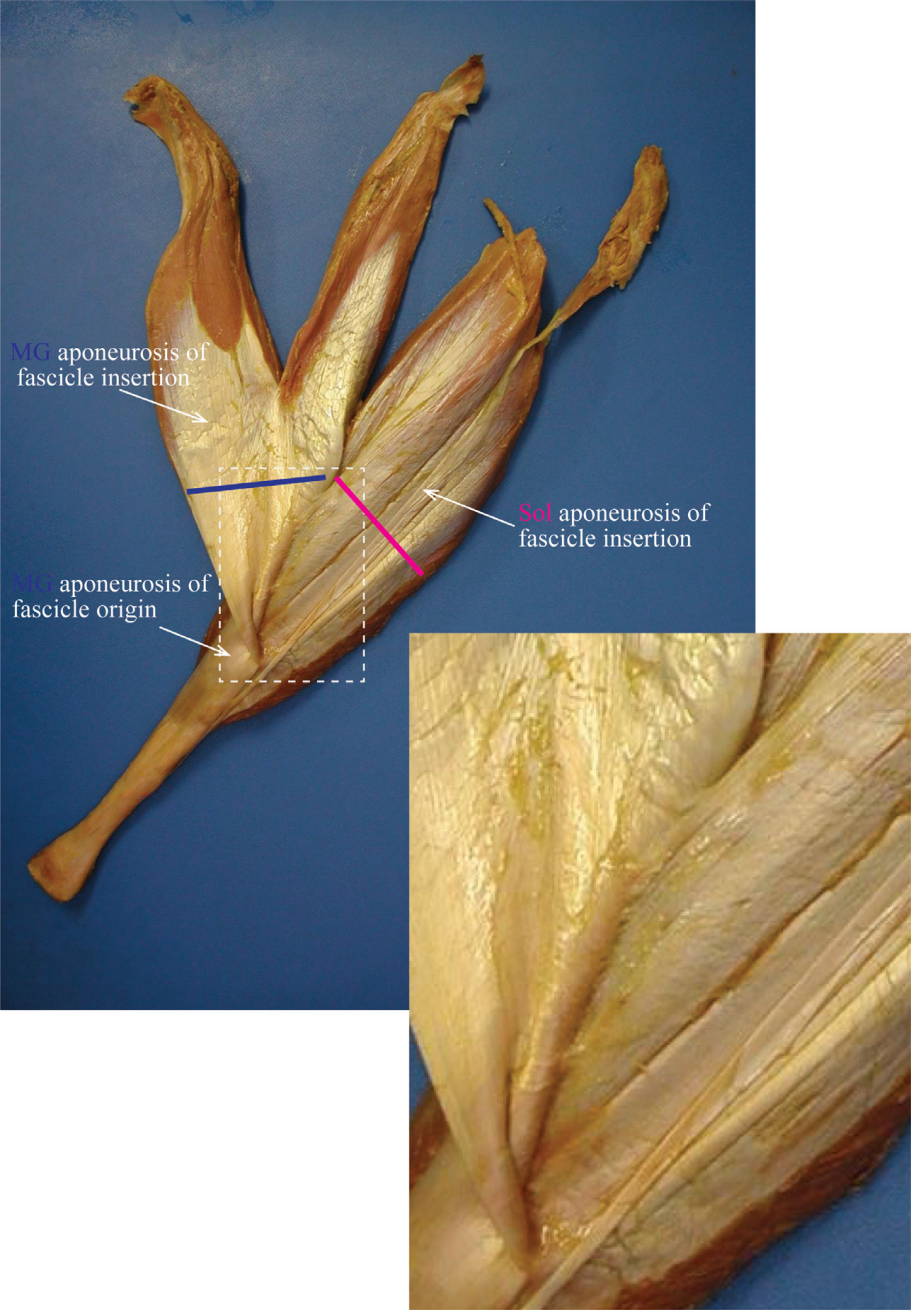
Medial view of a cadaver dissection of the gastrocnemius–soleus junction proximal to the Achilles tendon. Skin, subcutaneous tissue, and superficial fascia are removed. The gastrocnemius and soleus (Sol) muscle fibers are behind each aponeurosis. The gastrocnemius aponeurosis continues for a variable distance inferior to the distal ends of the medial and lateral heads of gastrocnemius to reach its line of attachment to the aponeurosis of the Sol fascicle insertion. The attachment between the aponeuroses of the medial gastrocnemius (MG) and Sol fascicles insertion enlarges on the bottom-right corner. Blue and pink lines correspond to locations where the myotendinous junction occurred and where the membrane thickness of the aponeurosis was measured using microscopy.

A digital microscope (VHX-2000; KEYENCE, Osaka, Japan) was used to determine the membrane thickness of the aponeurosis of fascicle insertion at its midportion along the entire length of the Sol and at the most distal edge of the MG (Fig. 1). The slice (∼10 mm × 10 mm) was dissected from the aponeurosis, and fat on the separated tissues was carefully cleaned. Each slice was then separated to eight pieces at regular intervals along the longitudinal direction. Each piece was nipped by pin set and placed on the slide glass without external tension.

### In vivo study

## Subjects

Eight healthy subjects (seven males and one female, age 30.1 ± 10.6 years, height 171.3 ± 7.7 cm, weight 71.4 ± 11.1 kg) with no prior history of muscle and tendon pathology participated in the investigation. Subjects were fully informed of the experimental protocol as well as the purpose of the study, and their informed consent to participate in the investigation was obtained. This study was approved by the ethical review board of University of California San Diego.

### Experimental setting and dynamometer

The subjects were positioned supine in the MRI scanner bore. The dominant leg (self-reported) was tightly fixed to a foot pedal dynamometer by using Velcro strap to minimize foot movement during joint movements. The subject was also secured to the bed with straps placed around the hips and knee with the knee joint at 180° to avoid movement. The axis of rotation of the dynamometer was aligned with the anatomical ankle dorsi- and plantarflexion axes. A custom-made dynamometer was programmed to complete 15° dorsiflexion to 30° plantarflexion with the cyclic period of 2.86 sec (21 cycles/min) using a computer-controlled servo motor-driven MR-compatible foot pedal device (Sinha et al. 2012). This specific range of motion (45°) was consistently applied to all subjects with minor adjustments to ensure the safety and comfort of each subject. The angular velocity was ∼31°/sec. A strain gauge based on an optical Fabry-Perot interferometer (Fiberscan 2000; Luna Innovations, Blacksburg, VA) was embedded in the sole of the foot pedal for measurement of the plantarflexion force. A signal conditioner, connected to the strain gauge with an optical cable, converted the measured strain signal proportional to the force exerted, into a digital signal, digitized at sampling frequency of 250 Hz (DAQ-6024E; National Instruments, Austin, TX). The target force level was set at 40% of the maximum voluntary contraction (MVC) during the plantarflexion phase of the foot rotation cycle, and was projected on the scanner face, allowing the subject to achieve the target forces production accurately and consistently. Forty percent MVC is about the maximum resistance at which the subject can sustain the ∼70 repeated contractions that are required for image acquisition without significant fatigue. The subject exerted 40% of MVC during the plantarflexion phase of the foot rotation cycle, thereby activating the plantarflexors under the shortening effect of the plantarflexion. The detail of dynamometer system and the reliability and validity of the dynamometer as well as lack of fatigue factor as evidenced by identical MVC before and after the MRI measurements have been reported in previous studies (Kinugasa et al. 2008; Sinha et al. 2012).

### Image acquisition

**Figure 2:**
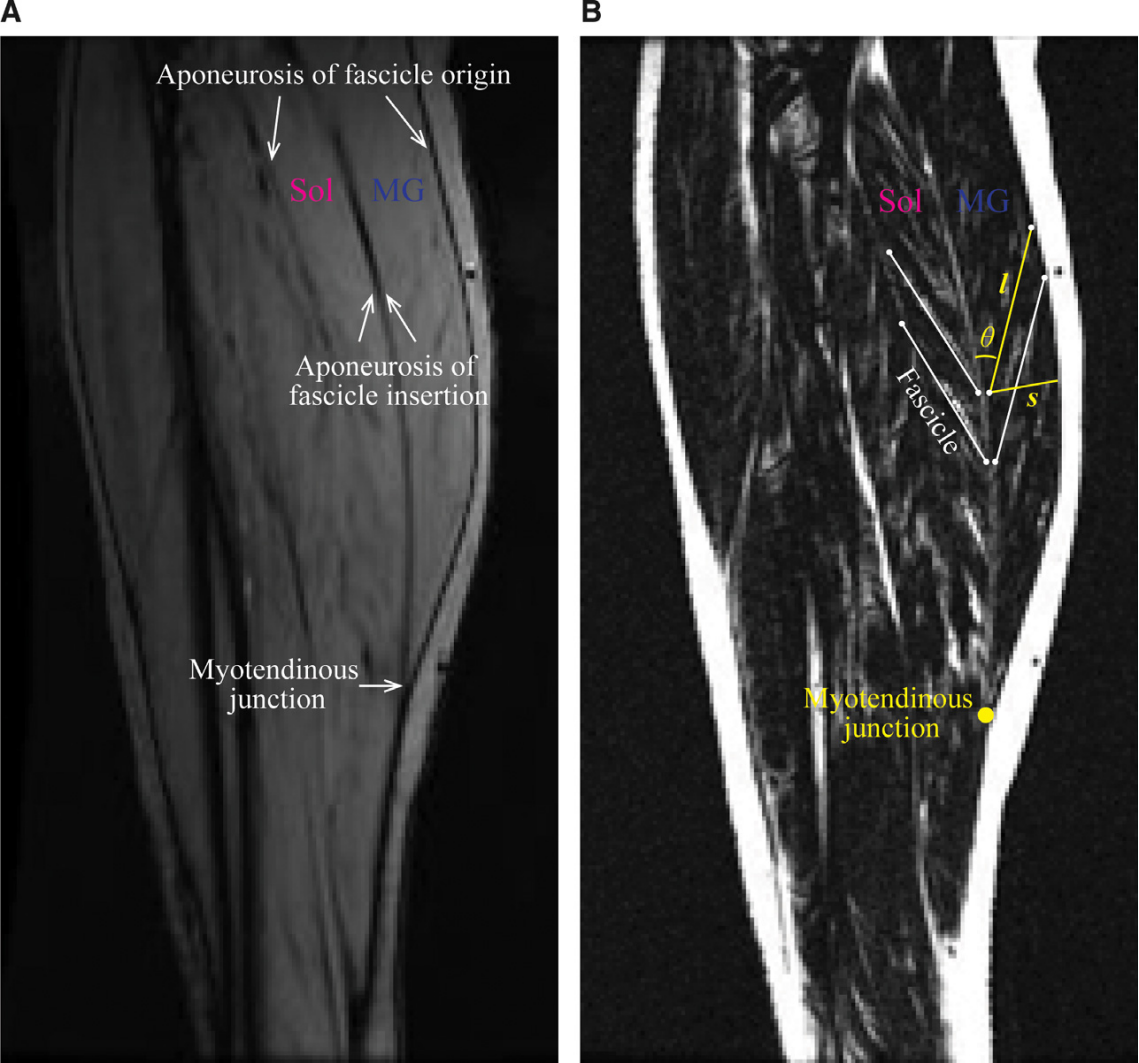
Representative magnitude image from a velocity-encoded, phase-contrast (PC) sequence (A), and water-saturated spin-lattice relaxation time (T1)-weighted image (B). The water-saturated T1-weighted image allowed one to identify the origin and insertion of the muscle fascicles, as shown by white dots. Those dots and the connecting lines were registered onto the PC magnitude images for displacement tracking of the fascicle and the subsequent calculation of fascicle length (*l*), pennation angle (*θ*), and thickness (*s*). The fascicle length was measured as the distance from the two ROIs identifying the fascicle origin and insertion points. The pennation angle was calculated as the angle between the line defined by the fascicle and the tangent at the aponeurosis at the point of fascicle insertion. The thickness was defined as the length of the normal from the point of fascicle insertion to the opposite aponeurosis.

A 1.5 T whole-body MR scanner (Signa HDx; GE Medical Systems, Milwaukee, WI) and a spine coil were used in all of the following image acquisitions. High-resolution static axial images of the calf were acquired using a gradient echo sequence (371 msec repetition time [TR], 2.4 msec echo time [TE], 45° flip angle [FA], 256 × 192-image matrix, 180 × 135-mm field of view [FOV], 5-mm slice thickness, 10-mm slice interval, bandwidth 244 Hz/pixel, one excitations, 25–35 slices, and 1:38 scan time). The axial images were then also used to specify an oblique sagittal plane, defined to bisect the MG, Sol, and aponeuroses, and oriented such that the aponeurosis was visualized in its entirety along the superior–inferior direction of the image (Fig. 2A). This plane allowed imaging the region in an orientation that best represented a section parallel to muscle fascicles of both the MG and Sol.

This oblique sagittal plane was first used to acquire a water-saturated spin-lattice relaxation time (T1)-weighted image, followed by the VE-PC scan. Imaging parameters for the water-saturated T1-weighted sequence were 2000 msec TR, 12.9 msec TE, 90° FA, 192 × 320-mm image matrix, 300 × 180-mm FOV, 3-mm slice thickness, 244 Hz/pixel bandwidth, three excitations, one slice, and 2:30 scan time. Water-saturated T1-weighted image was used to visualize fatty tissues running parallel to muscle fascicles and to determine the starts and ends of the muscle fascicles (Fig. 2B). The water-saturated T1-weighted scan was also acquired with the ankle at the plantarflexed position (120°). A VE-PC sequence (16.5 msec TR, 7.7 msec TE, 20º FA, 122 Hz/pixel bandwidth, 10 cm/sec velocity encoding in three directions, four views per segment, 22 phases, two excitations, 154 × 256-mm image matrix, 300 × 180-mm FOV, one slice, and 1:53 scan time) was then used to acquire tissue velocity-encoded dynamic images of the lower leg during ankle plantarflexion. VE-PC acquisition was synchronized to the foot rotational cycle. The output of the force transducer was electronically shaped to produce a pulse simulating an electrocardiographic R-wave at a particular threshold, triggering each segment of the phase encoding level to the beginning of the force rise.

### Image analysis

Images were analyzed using Matlab (The Mathworks Inc., Natick, MA). Prior to analysis, the 22 VE-PC images were first corrected for eddy current-induced spatial phase inhomogeneities by averaging the 22 images and subtracting it from each image, and smoothing images by 3 × 3 pixel averaging (Sinha et al. 2004). Regions of interest (ROIs) were then drawn as the origin and insertion point of the MG and Sol fascicles selected on the water-saturated T1-weighted image and then registered on to the VE-PC image. ROIs were positioned as close to the MTJ as possible, but it should be noted that ROIs identified here indicate accurately fascicle positions as reported previously (Maganaris et al. 1998). Both the MG and Sol fascicles insertion points were placed at similar horizontal levels and near MTJ (Fig. 2B). The velocities of the ROIs were obtained from the first VE-PC image. The positions in the subsequent VE-PC images were then estimated by the product of the velocity in the first VE-PC image and the time difference between the consecutive images (∼78 msec), thereby tracking the positions of the ROIs throughout the foot rotational cycle. This procedure allowed us to calculate the displacement (velocity × time) of the ROIs and to determine the displacement of aponeuroses on the MG and Sol sides, respectively. Care was taken to ensure that the projected ROI in the subsequent images did not fall into the aponeurosis region. The fascicle length ‘*l*’ was measured as the distance from the two ROIs identifying the fascicle origin and insertion points (Fig. 2B). The pennation angle ‘*θ*’ was calculated as the angle between the line defined by the fascicle and the tangent at the aponeurosis at the point of insertion of the fascicle. The thickness ‘*s*’ was defined as the length of the perpendicular from the point of insertion of the fascicle to the opposite aponeurosis. These aponeurosis displacement, fascicle length, fascicle angle, and thickness were quantified over 22 time phases. The ratio between fascicle length change and displacement of aponeurosis at fascicle insertion were used to calculate the gear ratio (Azizi and Brainerd 2008). The interaponeurosis shear strain was defined as the relative displacement of the aponeurosis of MG and Sol fascicles insertion, and calculated by dividing the MG displacement by the Sol displacement. The displacement of MTJ point was manually tracked from each magnitude image using ImageJ (National Institutes of Health, Bethesda, MD). Temporal difference of the displacement between the aponeurosis of the MG and Sol fascicles insertion was defined as the difference between the time at which the peak displacement of the MG and the Sol occurred.

### Statistics

All data are presented as means ± standard deviation (SD). The dependent variable for the gear ratio and aponeurosis displacement were compared with a one-factor (two muscles) analysis of variance (ANOVA). Correlation between two variables (Fig. 5) was assessed by determining Pearson's product-moment correlation coefficient. Significance was accepted at *P* < 0.05.

### Theoretical predictions of MTJ displacement

We determined theoretically how the magnitude of temporal difference impacts on the MTJ displacement, which was pulled by a component of the resultant displacement from each aponeurosis of the MG and Sol fascicles insertion. Theoretical MTJ displacement was calculated by the equation of equilibrium from the MG and Sol aponeuroses displacements (Appendix). Data were pooled for 16 variations of image frame (Table 1) at which maximum temporal difference occurred and each data point represents theoretical MTJ displacement over concentric phases (11 image frames) of the plantarflexion. The data were fitted to a second-order polynomial. The details of the methodology used for this theoretical modeling are described in Appendix.

**Table 1:** Effect of variation in image frames at which maximum temporal difference occurred and theoretical MTJ displacement

	Image frame when aponeurosis was displaced maximally			Temporal difference (sec)	Theoretical MTJ displacement (mm)

	Case	MG	Sol		
1	II	11	11	0.00	15.3
2		10	10	0.00	15.3
3		9	9	0.00	15.3
4		8	8	0.00	15.3
5		11	10	−0.14	14.6
6		11	9	−0.27	13.8
7	III	11	8	−0.41	13.1
8		10	9	−0.14	14.6
9		10	8	−0.14	13.7
10		9	8	−0.14	14.5
11		10	11	0.14	14.7
12		9	11	0.27	14.1
13	I	8	11	0.41	13.5
14		9	10	0.14	14.6
15		8	10	0.27	14.0
16		8	9	0.14	14.6

MG, medial gastrocnemius; MTJ, myotendinous junction; Sol, soleus.

## Results

### In vitro study

In the cadaveric specimen, the aponeuroses of the MG and Sol fascicles insertion were separate structures proximal to their common junction, but the two are joined by a thin membrane (Fig. 1). This in vitro observation indicated that the aponeurosis of the Sol fascicle insertion is attached to the MTJ. In an oblique sagittal PC magnitude image, connective tissue is depicted as a signal void or black color. The aponeuroses of MG fascicle origin and insertion and aponeurosis of the Sol fascicle insertion merged at the most distal edge of the MG, where they form a MTJ. There was no significant difference between the thickness of the two aponeuroses of MG and Sol fascicles insertion, with the membrane thickness for the MG being 621 ± 115 *μ*m and that for the Sol being 584 ± 91 *μ*m.

### In vivo study

**Figure 3:**
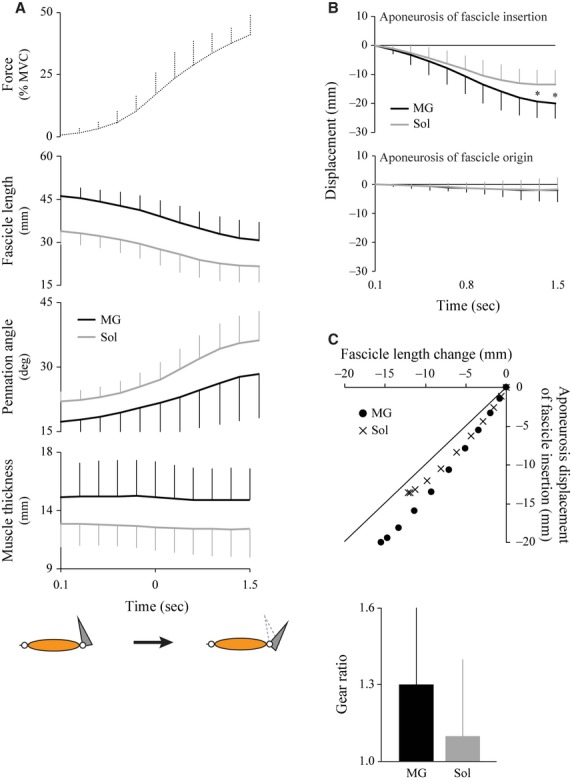
(A) Changes in force, fascicle length, pennation angle, thickness of the medial gastrocnemius (MG) (black), and soleus (Sol) (gray) muscles during concentric plantarflexion. Values are mean ± SD. Note that plantarflexion contraction was started at the dorsiflexed (15°) position of the ankle. (B) Changes in aponeurosis displacement of the MG (black) and Sol (gray) along the superior–inferior direction during concentric plantarflexion. The graph on the upper shows the aponeurosis of fascicle insertion and bottom shows the aponeurosis of fascicle origin. Values are mean ± SD. (C) The relationship between fascicle length change and displacement of aponeurosis at fascicle insertion along the axis of the MG (filled circle) and Sol (cross symbol). Each data point represents average value of eight individuals during concentric phase of the plantarflexion. The dotted black line is the line of identity where fascicle shortening and aponeurosis displacement are equal. The graph on the bottom shows that gear ratio of the fascicle length change to aponeurosis displacement along the axis of the MG (black) and Sol (gray). Values are means ± SD.

The lengths of both the MG and Sol fascicles shortened during concentric contraction (Fig. 3A). The pennation angle increased consistently throughout the period of contraction for both the muscles. The muscle thickness remained constant for both the muscles while the fascicles shortened. The behavior of the aponeurosis of fascicle insertion was different from that of the aponeurosis of fascicle origin (Fig. 3B). Locations below the zero line indicate movement in the proximal direction. Thus, the aponeuroses in both MG and Sol fascicles insertion moved the most in the proximal direction, whereas the aponeurosis of fascicle origin remained almost static or moved only slightly. In the aponeurosis of fascicle insertion, the MG has larger displacement than the Sol in the latter half of the concentric contraction (*P* < 0.05). The change in fascicle length was not identical to the displacement of the aponeurosis either for the MG or the Sol fascicle insertion (Fig. 3C, upper graph). The movement of the aponeurosis parallel to the long axis of the muscle exceeded or was amplifications of the shortening of the muscle fascicle. In other words, the gear ratio, which is defined as the mechanical advantage provided by the pennate muscle architecture wherein the aponeurosis movement is amplified per unit length change in fascicle, was greater than unity for both the MG and Sol, but not significantly different between them (Fig. 3C, bottom graph).

**Figure 4:**
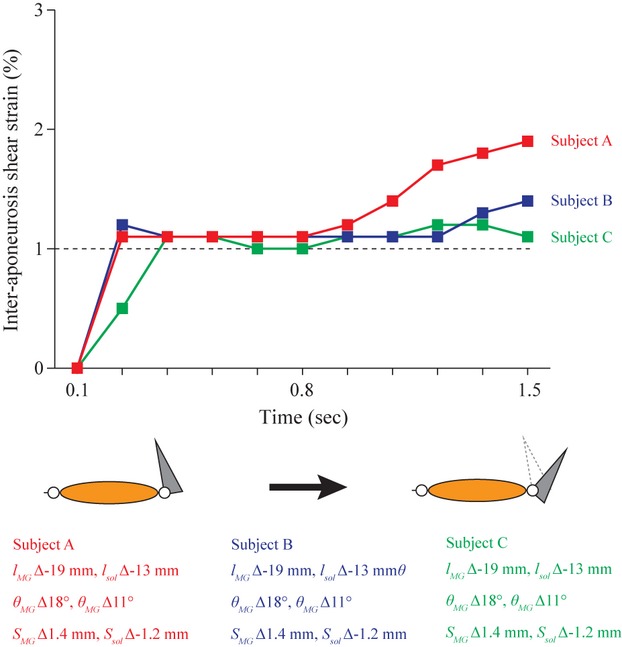
The interaponeurosis shear strain as a function of time during concentric plantarflexion in three subjects. A shear strain greater than unity indicated that the displacement of the aponeurosis in medial gastrocnemius (MG) fascicle insertion was greater than that of the soleus (Sol). A consistent pattern was observed over all subjects in that (Albracht et al. 2008) the peak value of shear strain occurred at the end of the concentric contraction for each subject, (Azizi and Brainerd 2008) a value of 1 for shear strain indicating similar aponeurosis displacement of the MG and Sol fascicles insertion occurred near the neutral position of the foot. Numerical values of fascicle lengths, pennation angle, and thickness are provided for the three subjects.

Interaponeurosis shear strain between the MG and Sol was determined at various values of aponeurosis displacement during different time points of the contraction cycle (Fig. 4). The magnitude of shear strain was an average of 1.37 ± 0.27% (range 1.12 ∼ 1.87%). Typically the shear strain was greater than unity, indicating that the displacement of aponeurosis at the MG fascicle insertion was greater than that of the Sol fascicle insertion. A peak value of shear strain was observed at the end of the concentric contraction for each subject. For example, Figure 4 indicates that while the peak value of interaponeurosis shear strain is not the same among the all subjects, the time points at which the peak values occur are, however, roughly at the same time period of the contraction cycle. The numerical values of change in fascicle length (*l*), pennation angle (*θ*), and thickness (*s*) of the MG and Sol are also indicated in Figure 4.

**Figure 5:**
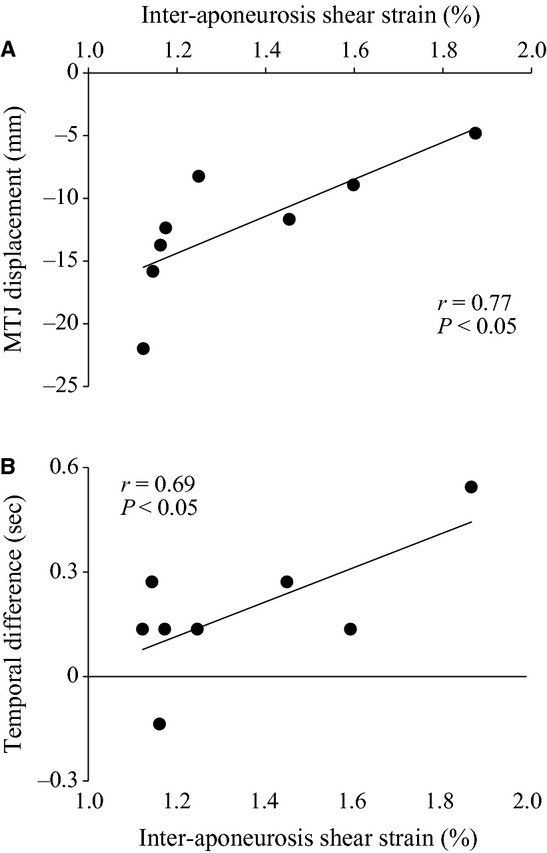
(A) The relationship between interaponeurosis shear strain (*x*-axis) and musculotendinous junction (MTJ) displacement (*y*-axis) (A), and interaponeurosis shear strain (*x*-axis) and temporal difference (*y*-axis) (B). Data pooled for eight individuals and each data point represents a value during the most plantarflexed ankle position. Temporal difference is calculated by subtraction of time phase at which peak displacement occurred in the aponeurosis of the medial gastrocnemius fascicle insertion from that of the soleus. A liner regression yields significant relationship between MTJ displacement and shear strain (*r* = 0.77, *P* < 0.05), and temporal difference and shear strain (*r* = 0.69, *P* < 0.05).

In order to determine how the MTJ displacement was influenced by interaponeurosis shear strain and temporal differences, we compared these three variables between individuals by plotting a linear regression. The temporal difference of the displacement of aponeurosis between MG and Sol fascicles insertion was defined by the difference between the time at which the peak displacement of the MG and the Sol occurred. MTJ displacement increased linearly with decreasing shear strain (Fig. 5A, *r* = 0.77, *P* < 0.05). There was also a significant correlation between shear strain and temporal difference (Fig. 5B, *r* = 0.69, *P* < 0.05). This pattern suggests that the interaponeurosis shear strain increased with increasing temporal difference.

### Theoretical predictions of MTJ displacement

**Figure 6:**
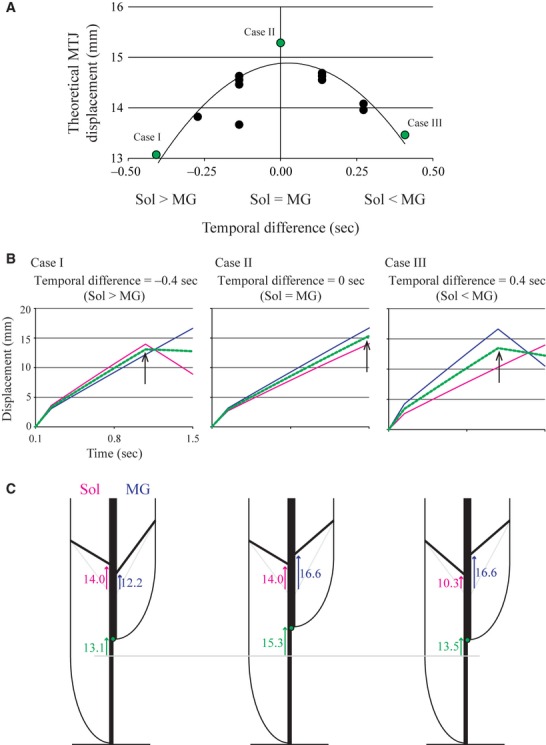
(A) The relationship between theoretical MTJ displacement and temporal difference. This was generated by various temporal differences resulting from different values of image frames at which aponeuroses displaced maximally (Table 1). Although the number of phase during concentric plantarflexion was 11 frames in this study, we varied the image frame where the medial gastrocnemius (MG) and soleus (Sol) aponeuroses indicated maximum displacement from 8 to 11. The temporal resolution of image frame set as 136 msec. The temporal difference when aponeuroses of MG and Sol fascicles insertion indicated maximum displacement impacted on the theoretical MTJ displacement curve over time in Figure A1 and consequently, it influenced the theoretical maximum value of MTJ displacement. However, note that if temporal difference is equal, theoretical maximum MTJ displacement is also equal. Rows #1, #7, and #13, which is indicated by pink lines, corresponds to Roman numeral as cases I, II, and III in Figure 6A. Negative temporal difference (*x*-axis) indicates the point at which the peak displacement of the aponeurosis in MG fascicle insertion occurred earlier than Sol. Fit to a second-order polynomial yielded *R*^2^ = 0.77, *P* < 0.05. The green circles (I, II, and III) correspond to representative MTJ displacement, which are indicated by the arrows, in each three graph in the (B). (B) Time-series plots of theoretical MTJ displacement (dashed green), aponeuroses of the MG (blue) and Sol (pink) fascicles insertion. Case II illustrates MTJ displacement when temporal difference between MG and Sol maximum displacement was zero (0 sec) while Cases I and III illustrates MTJ displacements for temporal differences of −0.4 sec and +0.4 sec, respectively. In all cases, the resultant displacement of the aponeurosis in MG and Sol fascicles insertion was consistent, but the time at which the peak displacement occurred changed. (C) A simplified diagram illustrating the effect of temporal difference on the possible movements of fascicle, aponeurosis, and MTJ during a shortening contraction. The three colored lines illustrate displacement of the aponeurosis in MG and Sol fascicles insertion, and MTJ (green, blue, and pink, respectively). The initial location of the fascicle and MTJ is shown in dotted line and open circle, respectively. The thickened black line and filled circles identify the fascicle length change, aponeurosis, and MTJ movements as the fiber shortens. The numbers indicate the aponeurosis displacement (in mm) of the MG (blue) and Sol (pink), and predicted MTJ displacement (green) as the arrow in each graph in (B). Horizontal gray line is the line of identity where the MTJ displacement is shown as maximum value among the three models (15.3 mm). See Appendix for details.

Figure 6 demonstrates the mathematical prediction of the dependence of theoretical MTJ displacement on the magnitude of the temporal difference calculated by the equation of equilibrium from the MG and Sol aponeuroses displacements (Appendix). The curve was best fit to the following parabolic function: *y* = −10.9*x*^2^ − 0.5*x* + 14.9, where *y* is the MTJ displacement, *x* is the temporal difference. The black-filled dots indicate the 16 variations of image frame at which maximum temporal difference occurred over the concentric phase, whereas the three green dots are representative time points at the maximum, zero, and negative maximum temporal difference between the MG and Sol displacements. The MTJ displacement was the largest (15.3 mm) when the temporal difference is zero, that is, when the peak displacement of aponeurosis both the MG and the Sol fascicles insertion occurred at the same time point. In contrast, a large temporal difference caused reduction in the MTJ displacement. For example, in case when the time at which the peak displacement of the MG occurred approximately 0.4 sec earlier than the Sol, which is the case of III, the MTJ displacement was reduced ∼12% to 13.5 mm. The displacement of the aponeurosis at MG fascicle insertion was then 16.6 mm, whereas the aponeurosis at Sol fascicle insertion was only 10.3 mm.

## Discussion

This study aims to understand the structural complexity of the human triceps surae complex. This combination of different muscle compartments and connective tissue acts together to flex the ankle and causes changes in localized displacement between adjacent aponeuroses to determine the overall displacement of the MTJ. The combination of experimental investigations and theoretical modeling presented here provides new generalized insights into the possible mechanisms of this combined action of adjacent muscles/aponeuroses proximal to the junction which connect them together. Our novel experimental finding illustrates an important relationship between interaponeurosis shear strain and mechanical properties of adjacent aponeuroses. Our modeling data presented here also clearly indicate that the MTJ behavior is influenced by the magnitude of the temporal difference between mechanical behaviors of different aponeuroses.

The MG muscle lies superficial in the posterior aspect of the lower leg with the independent insertion aponeurosis of the Sol muscle located anteriorly. Each muscle may be covered by thin epimysium which becomes broad aponeuroses of origin and insertion. The aponeurosis of the MG fascicle insertion runs a considerable distance over the Sol muscle proximal to the MG myotendinous junction. The aponeuroses of insertion of the two muscles, although lying adjacent to each other, only coalesce into a single common tendon distal to the myotendinous junction. This architectural arrangement constitutes a mechanism via which a shear strain can be produced between aponeuroses of the MG and Sol fascicles insertion (Bojsen-Møller et al. 2004). From our present observations of cadaver dissection experiments, we have confirmed that the aponeurosis of the MG and Sol fascicles insertion is joined by a thin membrane. Consequently, there was a loose connectivity between them which thereby allows the synchronous movement of one of the aponeurosis in fascicle insertion when the other one is pulled. In general, the aponeurosis of fascicle origin acts as the origin of the muscle, to which the proximal end of muscle fibers are attached, whereas the aponeurosis of fascicle insertion is the insertion of the muscle, with the distal end of muscle fibers attached to the MTJ for the MG or the Achilles tendon for the Sol. Therefore, both the aponeuroses of the MG and Sol fascicles insertion are in contact over the entire length of aponeurosis of the MG fascicle insertion. This study analyzed points of the muscle fascicles near the points of their insertion into the aponeurosis.

**Figure 7:**
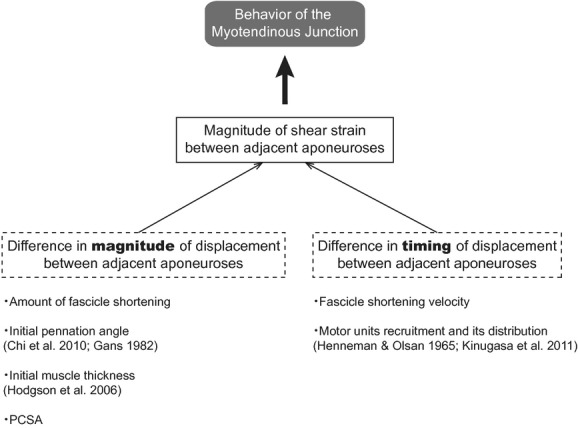
Schematic illustration of possible mechanism for the myotendinous junction (MTJ) behavior. The dotted black-lined boxes are the findings of this study. MTJ behavior is determined by the magnitude of individual aponeurosis displacement and magnitude of shear strain between adjacent aponeuroses (left dashed box). The former would be influenced positively by the aponeurosis behavior, but the latter would be negatively. The magnitude of shear strain is also determined by the temporal difference in aponeurosis displacement (right dashed box).

A shear strain of the aponeuroses occurring between the two muscles with a value of greater than unity implies that the extent of the displacement of the aponeurosis in MG fascicle insertion is greater than that of the Sol. All subjects exhibited not only such greater displacement of aponeurosis in the MG fascicle insertion compared to aponeurosis in the Sol fascicle insertion leading to values of shear strain greater than unity but also the occurrence of the peak value of the shear strain at the point of maximal contraction during the concentric phase (e.g., Fig. 4). The interaponeurosis shear strain is correlated with the timing difference in the displacement of the aponeurosis between the MG and Sol fascicles insertion (Fig. 5). The temporal pattern had a trend of a more rapid displacement of the MG compared to the Sol for most subjects, indicating that the peak displacement occurred earlier for the MG than for the Sol. The temporal difference is likely caused by differences in contraction times and fiber shortening velocity (Fig. 7) between the predominantly slow Sol and mixed fiber-type content of the MG (Edgerton et al. 1975). Based on this we can assume that peak shortening velocity of the fascicle during 40% of MVC was approximate 12.1 mm sec^−1^ for the MG and approximate 9.2 mm sec^−1^ for the Sol, which is calculated from changes in fascicle length over the time period (1.5 sec) of concentric contraction. Additionally, neuromuscular properties may also contribute to the temporal difference, in which recruitment of different types of motor unit is determined by the force production demanded (Henneman and Olsan 1965; Kinugasa et al. 2011) and subsequently the composition of the passive and active tissues within the muscle and tendon complex (Magnusson et al. 2003).

Besides the temporal pattern explained above, differences in magnitude of displacement between adjacent aponeuroses may also contribute to the interaponeurosis shear strain. It is widely acknowledged that the aponeuroses displacement is greater than the muscle fascicle shortening. This special mechanics of the muscle fiber–aponeurosis system has been explained based on the observation that the aponeuroses tend to move parallel to each other rather than along the direction of muscle fiber pull (Gans 1982). This ‘amplification’ of muscle fiber length changes into aponeurosis displacement has been termed the ‘gear effect’. The gain system may vary with the pennation angle as the instantaneous gain would be 1/cos (pennation angle) (Hodgson et al. 2006). We have also suggested that this architectural gear ratio is sensitive to changes in aponeurosis separation (Hodgson et al. 2006) as might occur during muscle atrophy, but in our case this factor is negligible, that is, the muscle thickness in Figure 3 remains constant. The relationship between the forces generated by the individual fibers to the aggregate force at the site of insertion, that is, the aponeurosis, is profoundly affected by the angle of pennation. As this angle increases, the useful vector, that is, displacement along the line of action (Gans 1982) decreases. Our previous simulations demonstrated that the local aponeurosis behavior in an isometric contraction is dependent on muscle fiber pennation angle, where aponeurosis shortening increases with smaller pennation angles (Chi et al. 2010). Consequently, the actual displacement at the aponeurosis would not only be affected by the contractile characteristics of the component sarcomeres but also by the complex geometry of the pinnate muscle design.

Elastic properties of tendinous tissue, which are generally determined by both the material and morphological properties, are probably a factor in the interaponeurosis shear strain. Our pilot cadaver dissection experiments showed that the membrane thickness of the MG and Sol aponeuroses of insertion were nearly identical, indicating that the dimensionally dependent elastic properties of the aponeurosis are probably not critical to the interaponeurosis shear strain. Finite element simulation suggested that the passive aponeurosis materials and nearby contracting muscle fibers behave as a composite material in which local mechanical properties may be quite different from the properties of the individual constituent materials (Chi et al. 2010). Thus, measures of aponeurosis behavior must be interpreted with caution. Other morphological factors such as the anatomical design of the Sol and distance it inserts onto the tendon could also determine the behavior of the interaponeurosis shear strain. Two distinct types of soleus–gastrocnemius junctions have previously been identified; the most common type involves the two structures contributing collagen fibers directly and equally to the Achilles tendon, while in an alternative arrangement the gastrocnemius aponeurosis inserts more proximally into the underlying Sol aponeurosis (O'Brien 1984; Kader et al. 2002). Such an anatomical design is subject to considerable variation between individuals. The structure and packing of the collagen fibers (Danielsen and Andreassen 1988) such as transverse bands of collagen fiber (Paavola et al. 2002), collagen fiber crimping (Patterson-Kane et al. 1997), and covalent intramuscular cross-links (Bailey 2001) are also generally considered to be factors affecting the materially dependent elastic properties.

To further verify the importance of the temporal difference of the MTJ behavior, we have investigated theoretically the relationship between the MTJ displacement and temporal difference between the time at which the peak aponeurosis displacement of the MG and Sol occurred. This is based on the mathematical predictions from the component of the resultant displacement of each aponeurosis of the MG and Sol fascicles insertion within several ranges of temporal differences. Theoretical MTJ displacement was the greatest when temporal difference was zero and small with high temporal difference (Fig. 6A). If the fascicle and aponeurosis do not move synchronously, MTJ displacement is consequently smaller with consequential effects upon force production.

It is interesting that most of the change in displacement of aponeurosis in the MG and Sol fascicles insertion and, consequently the highest interaponeurosis shear strain, occurred during the time of a concentric contraction, but not so in the aponeurosis of fascicle origin (Fig. 3B). For both the MG and Sol, these aponeuroses of fascicle insertion showed a progressive proximal movement, whereas the aponeuroses of fascicle origin were almost static or moved slightly. This is also in agreement with our previous observations (Kinugasa et al. 2008; Shin et al. 2009).

There is a distinct difference between our data presented herein and other reports in the literature. Oda et al. (2007) have reported that the behavior of MG and Sol fascicles during twitch contraction induced by an electrical nerve stimulus are almost identical. This inconsistency compared to our findings may arise from differences in either the nature of evoked contraction (voluntary vs. electrical stimulation) or its intensity (submaximal vs. supramaximal). However, our observation is supported by some relevant findings reported in the literature including intermuscle force transmission (Bojsen-Møller et al. 2010; Tian et al. 2012). Substantial changes in fascicle length have been observed in between the MG and Sol muscles when the MG is stimulated, while the Sol remains passive (Bojsen-Møller et al. 2010). Moreover, even when both muscles are passive, fascicle length changes in Sol can be observed during knee motion, implying that there is some force transmission, although the size of the transmitted force is likely to be small (Tian et al. 2012). Resolution of such conflicting observations needs to be further investigated before a proper understanding of muscle function can be realized.

Finally, the limitations of this study should be noted. First, we studied the MG and Sol muscles while other muscles such as lateral gastrocnemius, flexor halluces longus, tibialis posterior, or flexor digitorum longus muscles also contribute to MTJ displacement and the Achilles tendon force. Uneven shortening of separate triceps surae muscles have been observed by estimating changes in muscle length from measurements of pennation angle and fascicle length at various knee and ankle joint angles (Kawakami et al. 1998). Consequently, the behavior of these other muscles during plantarflexion–dorsiflexion contraction might be different from that of the MG and Sol muscles. The effect of these other muscles on MTJ displacement and ultimately tendon forces needs to be tested in future studies. Second, the fact that the relative force produced by different muscle is unknown. Therefore, the predictions we have developed are based on the assumption that the contribution of each muscle to the movement of the MTJ is equal and can be averaged based on strains. However, this assumption does not take into account the fact that the displacement of the tendon is ultimately a function of a force and that differences in forces between the two muscles can be decoupled producing shear strain in the aponeurosis of insertion and the displacement of the MTJ. Thus, the question arises: how would the variation in the force capacity of the different muscles, in contrast to the equal contribution our model presumed, alter our predictions? The physiological cross-sectional area (PCSA) was not similar between the MG and Sol, with that of the MG being about 2 ∼ 3 times greater in the Sol (Fukunaga et al. 1997; Albracht et al. 2008). During a submaximal 40% MVC, uncertainty regarding the force produced by each muscle is compounded further, because there are differences in recruitment strategies between two muscles (Nardone and Schieppati 1988; Nardone et al. 1989; Kinugasa et al. 2011).

In conclusion, the results presented here highlight a previously unappreciated mechanism of the MTJ behavior that is caused by events at the fascicle level in pinnate muscles. The main finding is that points on adjacent surfaces of the two muscles are displaced relative to each other during the contractions, and that the largest distal displacement of the myotendinous junction is observed in those subjects whose MG and Sol muscles experience peak displacements at approximately the same time. Our findings also suggest that the synchronized behavior of the two muscle compartments rendered structurally independent by the tendinous tissue and ultimately connective tissue is a critical and unique feature of the system, which facilitates movement of the tendinous tissue and improves force output during muscle contraction. Such studies, particularly when extended to activation studies with electromyogram of other muscle compartments that take part in the ankle plantarflexion may have important implications for connective tissue injury and fiber-type-specific atrophy in aged skeletal muscle.

## Figures and Tables

**Figure A1:**
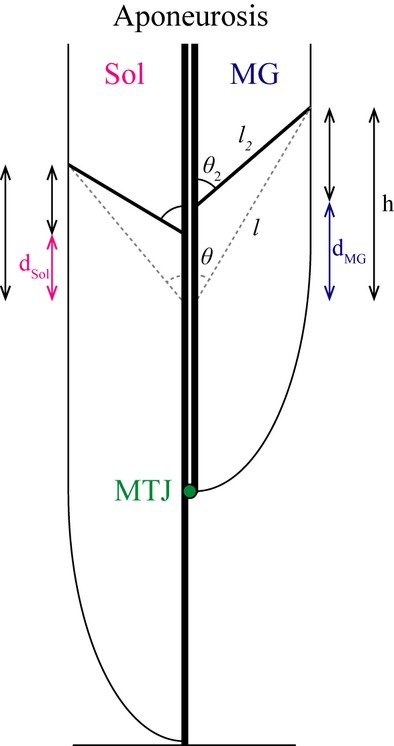
The model used to calculate the relationship between the displacement of myotendinous junction (MTJ) and aponeurosis displacement for the medial gastrocnemius (MG) and soleus (Sol) fascicles insertion.

## Appendix

### Calculation of theoretical MTJ displacement (Fig. A1)

For a given rest length of muscle fascicle (*l*) and pennation angle (*θ*):

The height projected by the fascicle onto the aponeurosis (h) = *l* * cos (*θ*).

If the fascicle length is shortened to *l*_2_, new pennation angle is then *θ*_2_. New height projected by the fascicle shortening onto the aponeurosis (h_2_) is: *l*_2_ * cos (*θ*_2_). Therefore, the displacement of the aponeurosis (d) is: h − h_2_.

Figure A1 was generated by modeling a MG fascicle shortening to 24.1 mm and pennation angle increasing to 39.0° from a rest fascicle length of 39.5 mm and a pennation angle of 22.6°. For the Sol, fascicle shortening to 18.4 mm and pennation angle increasing to 41.0° from a rest fascicle length of 29.8 mm and a pennation angle of 24.6°. These numerical characteristics were made from the present experimental observations. Aponeurosis separation was assumed to remain constant during fascicle shortening.

For a given aponeurosis displacement for the MG (d_MG_) and Sol (d_Sol_), the displacement of myotendinous junction (d_MTJ_), and spring constant (k):

For the aponeurosis extension:

(A1)

(A2)

For the aponeurosis force:

(A3)

(A4)

For the resultant force:

(A5)

where F is a force in the inferior direction. However, F is assumed to be zero since the inferior MTJ, tendon force is not involved. Consequently, renewed modified equation should be considered as follows.

(A6)

MTJ displacement is then calculated as:

(A7)
